# Association between socioeconomic factors and unmet need for modern contraception among the young married women: A comparative study across the low- and lower-middle-income countries of Asia and Sub-Saharan Africa

**DOI:** 10.1371/journal.pgph.0000731

**Published:** 2022-07-27

**Authors:** Asibul Islam Anik, Md Rashedul Islam, Md Shafiur Rahman

**Affiliations:** 1 Department of Population Science and Human Resource Development, University of Rajshahi, Rajshahi, Bangladesh; 2 Hitotsubashi Institute for Advanced Study, Hitotsubashi University, Tokyo, Japan; 3 Division of Prevention, Institute for Cancer Control, National Cancer Center, Tokyo, Japan; 4 Research Center for Child Mental Development, Hamamatsu University School of Medicine, Hamamatsu, Japan; 5 United Graduate School of Child Development, Osaka University, Kanazawa University, Hamamatsu University School of Medicine, Chiba University and University of Fukui, Hamamatsu, Japan; PLOS: Public Library of Science, UNITED STATES

## Abstract

Modern contraceptive methods are effective tools for controlling fertility and reducing unwanted pregnancies. Yet, the unmet need for modern contraception (UNMC) remains high in most of the developing countries of the world. This study aimed to compare the coverage of modern contraceptive usage and the UNMC among the young married women of low- and lower-middle-income countries (LMICs) of Asia and Sub-Saharan Africa, and further examined the likelihood of UNMC across these regions. This cross-sectional study used Demographic and Health Survey (DHS) data on family planning from 32 LMICs of South Asia (SA), Southeast Asia (SEA), West-Central Africa (WCA), and Eastern-Southern Africa (ESA). Multilevel logistic regression models were used to investigate the relationship between UNMC and women’s socioeconomic status. Out of 1,00,666 younger married women (15–24 years old), approximately 37% used modern contraceptives, and 24% experienced UNMC. Regionally, women from SA reported higher modern contraceptive usage (44.7%) and higher UNMC (24.6%). Socioeconomic factors like- higher education (in SA and WCA), unemployment (in SA and ESA), no media exposure (in SA and ESA), and higher decision-making autonomy (except SEA) showed positive and significant association with UNMC. Poorest households were positively associated with UNMC in SA and ESA, while negatively associated with UNMC in SEA. UNMC was highly reported among the SA young married women, followed by WCA, SEA, and ESA regions. Based on this study findings, versatile policies, couples counseling campaigns, and community-based outreach initiatives might be undertaken to minimize UNMC among young married women in LMICs.

## 1. Introduction

Unwanted pregnancies and unsafe abortions can seriously affect any sexually active women and have negative impacts on women’s personal and conjugal life, their families, and societies. Due to unsafe abortions, thousands of women die and millions more suffer long-term reproductive problems, including infertility. The incidence of unwanted pregnancies and unsafe abortions is likely to continue to increase until women’s need for modern contraception is met [1]. To estimate women’s need for family planning services (i.e., modern contraception) and assess women’s ability to obtain their reproductive desire, recently the concept ‘unmet need for modern contraception’ (UNMC) has been introduced [2]. Globally this important tool is widely used for advocacy, developing family planning policies, and implementing and/or monitoring family planning programs [3]. Conceptually, UNMC captures those sexually active or fecund women who are not using modern contraceptive tools but intend to conceive a child later, or to abstain of having any more children [3,4]. Since the degree of UNMC is one of the basic indicators for evaluating the effectiveness of family planning program in any country, women having UNMC are the logically important targets for such program management [3,4].

In 2012, the global community launched the Family Planning 2020 (FP2020) initiative at ‘London Summit for Family Planning’ which is built on the principle that all women, regardless of their place of residence and economic status, should enjoy their human right to access safe and effective, voluntary contraceptive services and commodities [4]. Since then, the FP2020 movement has focused on 69 poorest countries, and consequently, the global coverage of modern contraceptives among reproductive age married women has been increased by 30.2 million from 2012 (270 million) to 2016 (more than 300 million) [5]. However, the usage of modern contraceptives among married women was increased slowly in Asia (from 51 percent to 51.8 percent) between 2012 to 2017, compared to their counterparts in the African region (from 23.9 percent to 28.5 percent) [6]. On the other hand, the overall UNMC was reported to be 21.6 percent among the FP2020 focused countries in 2017 with a coverage of over 25 percentage in most of the Southern Asian and Sub-Saharan African countries [6]. This higher percentage of UNMC indicate a significant barrier in achieving Sustainable Development Goal 3.7 (SDG-3.7). The high dominance of UNMC backpedals the achievement of a higher proportion of demand satisfied by modern methods, which is one of the major health related indicators of the SDG (SDG Indicator 3.7.1) [5,7].

Efforts to reduce the extent of UNMC effectively require the region-wise assessment of the socio-demographic characteristics of the population and the identification of underlying factors that directly influence unmet needs [8,9]. Though some country-specific studies [8,10,11] and much earlier literature [2,12–14] reported that different socio-economic factors of women; limited choice and access to family planning methods; fear of side effects of using contraceptives; child marriage; urban-rural disparities; spousal age difference; and religious or cultural constraints, etc. have the potentiality to shape the level of UNMC among reproductive-aged women [8,11,12,15]. Moreover, some of these studies included all sexually active women regardless of their age and marital status [6,15]. But compared to the older married women, it has been reported that young married women (aged 15–24 years old) experience disproportionately higher levels of UNMC owing to their distinct fertility preferences, (i.e., partner’s desire of more or male children, avoiding older-aged pregnancy complications, and persistent high child mortality, etc.), and such preferences varied from culture-to-culture [2,12,13,15]. Hence, the actual association between different socio-economic factors and UNMC, especially for the younger married women, might not be reflected properly after including all reproductive aged women.

The percentage of UNMC is still high among the younger married women of low- and lower-middle-income countries (LMICs), particularly from South Asian, Southeast Asian, and Sub-Saharan African region [2]. But rarely any study has explored and compared the prevalence and associated factors of UNMC among the young married women of these regions. Though Ahinkorah et al. (2020) [16] investigated on the socio-demographic variations in unmet need for contraception among the younger aged women, that study was conducted regardless the marital status, confined to only Sub-Saharan African region, and considered any types of contraception. Such limitations of the existing literature impede international comparability and manifest the necessity of region-by-region investigation of UNMC and its associated socioeconomic factors among the young married women of LMICs. On the other hand, a comprehensive comparative research examining the present prevalence of UNMC and identifying the associated factors will assist policymakers in the individual regions to adapt and implement successful family planning programs based on their respective cultural contexts and the socioeconomic factors. Aiming these issues, this comparative study investigated the coverage of modern contraceptive usage and UNMC among the young married women, and further identified the socioeconomic factors that were associated with UNMC in the LMICs of Asian and Sub-Saharan African regions.

## 2. Methods

### 2.1 Data sources

Data from latest Demographic and Health Surveys (DHS) with available information on family planning conducted in 32 LMICs of Southern Asia and Sub-Saharan Africa (from 2014 onwards) were used. Five countries from South Asia (Afghanistan, Bangladesh, India, Nepal, and Pakistan), four from Southeast Asia (Cambodia, Myanmar, Philippines, and Timor-Leste), 13 from West and Central Africa (Angola, Benin, Cameroon, Chad, Congo Democratic Republic, Ghana, Guinea, Liberia, Mali, Nigeria, Senegal, Sierra Leon, and Togo), and 10 countries from East and Southern Africa (Burundi, Ethiopia, Kenya, Lesotho, Malawi, Rwanda, Tanzania, Uganda, Zambia, and Zimbabwe) were included. DHS is publicly available, nationally representative, cross-sectional surveys conducted in LMICs with multistage (usually two-stage) cluster sampling. Along with other information on maternal and child health outcomes and interventions, DHS regularly gather information on family planning and reproductive health. However, detailed administrative procedures, trainings, sampling strategies and methodology of DHS have been described elsewhere [17,18].

### 2.2 Study population

This cross-sectional study was limited to only currently married younger women aged 15–24 years old. After excluding the missing information on outcomes or covariates, a total of 100,666 married women, with complete information from 32 LMICs of Asia and Sub-Saharan Africa (SSA), were finally selected for this study (Table 1).

**Table 1:** Number of currently married younger women with complete information.

Country	Year	Phase	No. of household interviewed	No of women interviewed (15–49 years)	Currently married younger women (15–24 years)	Missing	Total sample
**South Asia (n = 55,953)**							
Afghanistan	2015	DHS VII	24,395	29,461	7,840	62	7,778
Bangladesh	2017–18	DHS VII	19,457	20,127	5,441	1	5,440
India	2015–16	DHS VII	6,01,509	6,99,686	98,767	60,866*	37,901
Nepal	2016	DHS VII	11,040	12,862	2,387	0	2,387
Pakistan	2017–18	DHS VII	11,869	12,364	2,447	0	2,447
**Total**			**6,68,270**	**7,74,500**	**1,16,882**	**60,929**	**55,953**
**Southeast Asia (n = 4,937)**							
Cambodia	2014	DHS VII	15,825	17,578	2,252	1	2,251
Myanmar	2015–16	DHS VII	12,500	12,885	1,061	4	1,057
Philippines	2017	DHS VII	27,496	25,074	702	0	702
Timor-Leste	2016	DHS VII	11,502	12,607	927	0	927
**Total**			**67,323**	**68,144**	**4,942**	**5**	**4,937**
**West and Central Africa (n = 23,491)**							
Angola	2015–16	DHS VII	16,109	14,379	304	0	304
Benin	2017–18	DHS VII	14,156	15,928	2,035	0	2,035
Cameroon	2018	DHS VII	11,710	13,527	1,281	0	1,281
Chad	2014–15	DHS VII	17,233	17,719	3,443	9	3,434
Congo DR	2013–14	DHS VI	18,171	18,827	2,003	3	2,000
Ghana	2014	DHS VII	11,835	9,396	350	0	350
Guinea	2018	DHS VII	7,912	10,874	1,773	0	1,773
Liberia	2019–20	DHS VII	9,068	8,065	234	0	234
Mali	2018	DHS VII	9,510	10,519	2,378	0	2,378
Nigeria	2018	DHS VII	40,427	41,821	6,025	23	6,002
Senegal	2019	DHS VIII	4,538	8,649	1,335	4	1,331
Sierra Leon	2019	DHS VII	13,399	15,574	1,602	0	1,602
Togo	2013–14	DHS VI	9,549	9,480	768	1	767
**Total**			**25,568**	**1,85,278**	**23,531**	**40**	**23,491**
**Eastern and Southern Africa (n = 16,285)**							
Burundi	2016–17	DHS VII	15,977	17,269	964	0	964
Ethiopia	2016	DHS VII	16,650	15,683	2,224	0	2,224
Kenya	2014	DHS VII	36,430	31,079	3,338	1,785^¥^	1,552
Lesotho	2014	DHS VII	9,402	6,621	942	0	942
Malawi	2015–16	DHS VII	26,361	24,562	4,492	0	4,492
Rwanda	2014–15	DHS VII	12,699	13,497	281	0	281
Tanzania	2015–16	DHS VII	12,563	13,266	1,415	1	1,414
Uganda	2016	DHS VII	19,588	18,506	1,205	0	1,205
Zambia	2018	DHS VII	12,831	13,683	1,862	0	1,862
Zimbabwe	2015	DHS VII	10,534	9,955	1,348	0	1,348
**Total**			**1,73,035**	**1,64,121**	**18,071**	**1,786**	**16,285**
**Pooled total (N)**							**1,00,666**

Note: n, total number of respondents of each region; N, pooled sample size from four regions.

*In India, about 59,369 respondents did not respond the questions regarding ‘decision-making’ at household level.

^¥^In Kenya, 1785 respondents did not respond the question regarding ‘hearing family planning message on mass media’.

### 2.3 Measurements

#### 2.3.1 Outcome variable

The outcomes of interest were modern contraceptive usage and UNMC.

*Modern contraception methods*. Modern contraception methods include contraceptive pills, condoms (male and female), intrauterine device (IUD), injectables, hormone implants, sterilization (male and female), patches, diaphragms, spermicidal agents, and emergency contraception [17]. Prevalence of modern contraceptive usage was determined as the percentage of women of reproductive age who report themselves or their partners as currently using at least one of the modern contraception methods.

*Unmet need for modern contraception*. UNMC, the third core indicator of FP2020 initiative, was measured as the percentage of fecund women of reproductive age who want no more children or to postpone having the next child, but are not using any contraceptive method, plus women currently using a traditional method of family planning [3]. Women using any of the traditional methods (like- abstinence, the withdrawal method, the rhythm method, douching, and folk methods) were also assumed to have a UNMC. Again, pregnant women with a mistimed or unwanted pregnancy were also considered in need of contraception.

#### 2.3.2 Exposures

Based on the empirical literature [8,9,19,20], this study considered four variables i.e., educational level, type of earning from work, exposure to media (family planning messages), and household level decision-making autonomy—as the proxy variables to indicate the socioeconomic status of respondents. Educational level was classified as no education, primary, secondary, and higher. Type of earning from respondent’s works was categorized into not working, working and paid (cash paid, or in-kind paid, or both), and working but not paid. Exposure to family planning messages via mass media refers to hearing family planning messages via listening radio, watching TV, and reading newspaper for the last few months. ‘Exposure to media’ was dichotomized by assigning a value of 1 if the respondent heard family planning messages from at least one of the mass media, and 0 if they did not. Women’s household-level decision-making autonomy was measured using their responses to four questions that asked who makes decisions in the household regarding obtaining health care for herself, making large purchases, visiting family and relatives, and using contraception. Response categories were the respondent alone, the respondent and her husband/partner jointly, her husband/partner alone, someone else or other. For each of the four questions, a value of 1 was assigned if the respondent was involved in making the decision, and 0 if she was not; then, the values were summed and dichotomized as ‘participated’ and ‘not participated’. And finally, the household wealth index, which was measured by the DHS authority using principal component analysis of the assets owned by households, and the detailed analytical procedures were described elsewhere [21]. The score was categorized into five equal quintiles (poorest, poorer, middle, richer, and richest) with the first, representing the poorest 20%, and the fifth, representing the richest 20%.

#### 2.3.3 Controlling variable

Based on the previous studies [8,9,11,12,15], the following controlling variables were used in the analyses along with the predictor variables: partner was more educated than the wife (yes, no); spousal age difference (less than 5 years, 5 to 9 years, 10 years and more); the number of living children (no child, 1 to 3, 4 and more); whether respondents married before 18 years old (yes, no); and place of residence (urban, rural).

### 2.4 Statistical analysis

Frequency distribution and univariate analysis were used to compare the proportion of UNMC with the socio-economic status of respondents. Multilevel logistic regression models with a random intercept term at community- and country-level were used to estimate adjusted odds ratios (ORs), along with 95% confidence intervals (CIs), for the relationship between exposures and UNMC. Models were adjusted for- partner was more educated than the wife; spousal age difference; number of living children; marriage before 18 years old; and place of residence. For all analyses, P < 0.05 was set as the significant level. The complex survey (DHS) design was considered in all the analyses using Stata’s ’SVY’ command. Data management and statistical analysis were conducted in Stata version 16.1/MP.

## 3. Result

### 3.1 Country-specific coverage of modern contraceptive and percentage of unmet need

Overall, 1,00,666 young married women from Asia and Sub-Saharan Africa were included in this analysis. The mean age (±SD) of the study population was 21.17 (±2.23) years, while their mean age (±SD) at marriage was 17.15 (±2.50) years. The pooled estimate from 32 LMICs showed that about 37% young married women used modern contraceptives and 24% women had UNMC (S1 Table).

**Fig 1:**
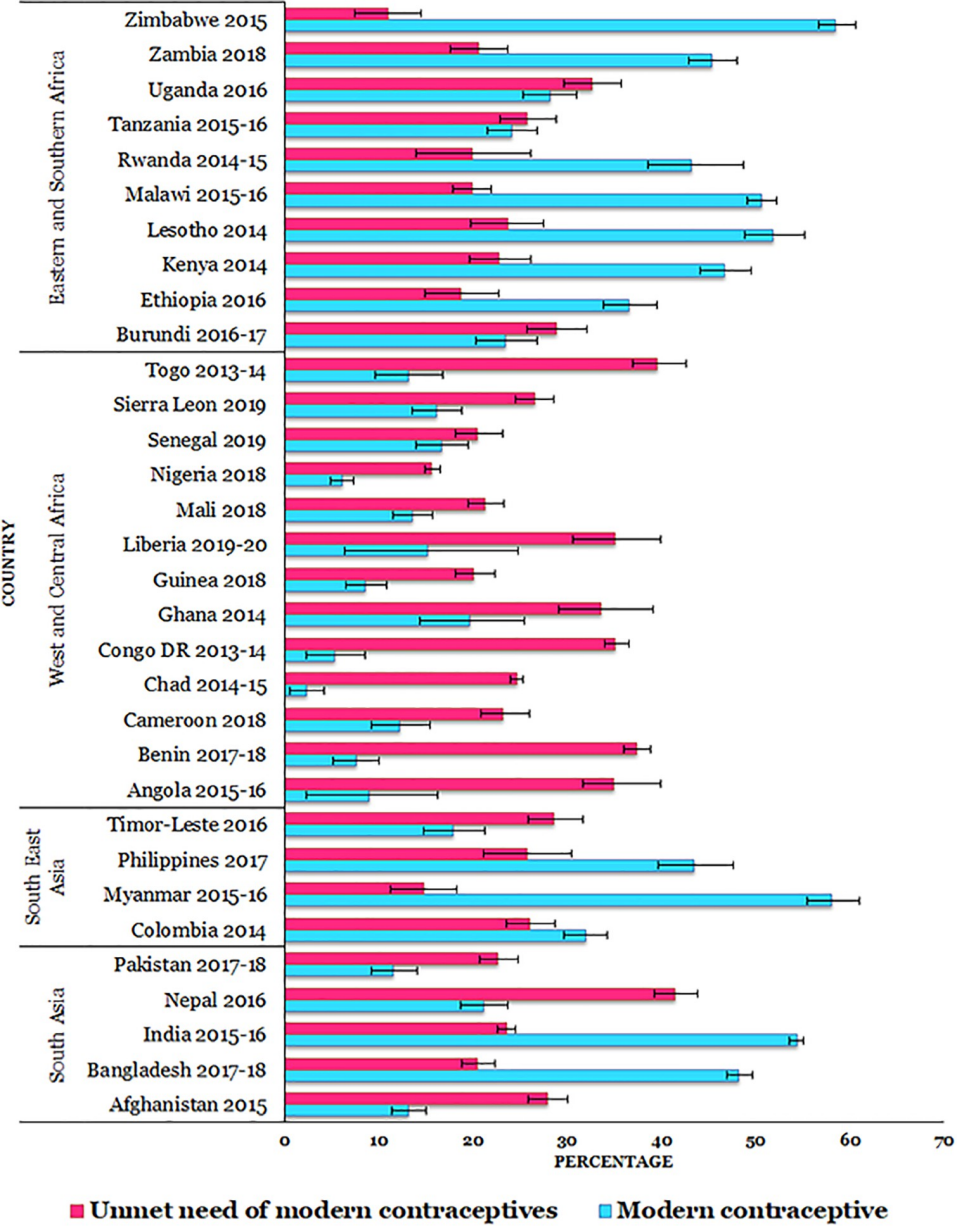
Percentage of modern contraception usage and unmet need of modern contraception in Asia and sub-Saharan Africa. Note: Error bar presents 95% confidence intervals.

From the country specific estimation, the overall percentage of modern contraceptive usage was higher in South Asia (SA) (44.7%; CI: 43.9% - 45.6%), followed by Eastern and Southern Africa (ESA) (42.7%; CI: 41.6% - 43.8%), and Southeast Asia (SEA) (36.5%; CI: 34.8% - 38.3%) (S1 Table). Modern contraceptive usage varied from 11.6% (Pakistan) to 54.4% (India) in SA, from 17.9% (Timor-Leste) to 58.1% (Myanmar) in SEA, from 2.3% (Chad) to 19.7% (Ghana) in WCA, and from 23.5% (Burundi) to 58.5% (Zimbabwe) in ESA (Fig 1).

On the other hand, the UNMC was highly reported in SA (24.6%; CI: 24.0% - 25.1%), compared to WCA (24.2%; CI: 23.5% - 25.0%), SEA (24.0%; CI: 22.6% - 25.5%), and ESA (21.5%; CI: 20.7% - 22.4%) (S1 Table). The proportion of UNMC ranged from 20.5% (Bangladesh) to 41.5% (Nepal) in SA, from 14.8% (Myanmar) to 28.5% (Timor-Leste) in SEA, from 15.6% (Nigeria) to 39.5% (Togo) in WCA, and from 11.0% (Zimbabwe) to 32.6% (Uganda) in ESA (Fig 1).

The younger married women of Asia possessed higher socioeconomic status in all the selected aspects than their counterparts of SSA. The percentage distribution of different socio-economic characteristics of the study population has been displayed in S2 Table. On the other hand, S3 Table showed that the UNMC was significantly higher among the South Asian younger women who had not-paid job (29.1% vs 22.4%), no media exposure (25.4% vs 23.9%), and high decision-making autonomy (27.5% vs 23.0%) than those with paid job, media exposure, and medium decision-making autonomy. In contrast, women from East and South Africa who received secondary and higher level (17.5% vs 24.6%), possessed medium decision-making power (18.7% vs 39.7%), and richest wealth-index (17.2% vs 24.6%) reported significantly lower proportion of UNMC compared to those with no education, high decision-making autonomy, and poorest household (S3 Table).

### 3.2 Socioeconomic factors affecting unmet need for modern contraception

To investigate the associated socioeconomic factors for UNMC, the models (unadjusted and adjusted) of multilevel logistic regression analyses from the four regions and the pooled data have been presented in Tables 2 and *S4*, respectively. From the adjusted analysis (Model II), SA countries showed that women’s secondary and higher education level [AOR: 1.37; 95% CI: 1.26–1.49], not working status [AOR: 1.23; 95% CI: 1.13–1.33], no media exposure [AOR: 1.12; 95% CI: 1.06–1.18], high decision-making autonomy [AOR: 1.30; 95% CI: 1.15–1.46], and poorest wealth index [AOR: 1.15; 95% CI: 1.05–1.26] showed positive and significant association with UNMC, after adjusting potential covariates. For SEA countries, women’s medium decision-making autonomy and poorest wealth index showed 21% and 38% lower likelihood of experiencing UNMC than their counterparts with low decision-making autonomy and richest wealth index, respectively. On the other hand, socio-economic factors like primary education [AOR: 1.26; 95% CI: 1.16–1.38], secondary and higher education [AOR: 1.50; 95% CI: 1.36–1.65], and high decision-making autonomy [AOR: 1.35; 95% CI: 1.09–1.66] were positively and significantly associated with UNMC among the women of WCA. In ESA, while women with high decision-making autonomy had 1.94 times higher odds, women’s medium level decision making had 14% lower odds of experiencing UNMC, compared to the women with low decision-making autonomy. Additionally, women’s not working status, no media exposure, and poorest wealth index reported positive and significant association with UNMC in ESA (Table 2).

**Table 2:** Association of socio-economic factors with unmet need for modern contraceptives in 30 low- and middle-income countries.

Socio-economic factors	Odds ratio (95% CI)							
	South Asia (N = 55,953)		Southeast Asia (N = 4,937)		West and Central Africa (N = 23,491)		East and Southern Africa (N = 16,285)	
	Model I	Model II	Model I	Model II	Model I	Model II	Model I	Model II
**Educational level**								
No education	***1*.*00 (Ref*.*)***	***1*.*00 (Ref*.*)***	***1*.*00 (Ref*.*)***	***1*.*00 (Ref*.*)***	***1*.*00 (Ref*.*)***	***1*.*00 (Ref*.*)***	***1*.*00 (Ref*.*)***	***1*.*00 (Ref*.*)***
Primary	1.00 (0.92–1.08)	1.01 (0.94–1.09)	1.07 (0.81–1.42)	1.10 (0.83–1.47)	1.30*** (1.19–1.41)	1.26*** (1.16–1.38)	1.03 (0.91–1.17)	1.10 (0.96–1.26)
Secondary and higher	1.20*** (1.13–1.28)	1.37*** (1.26–1.49)	1.12 (0.85–1.47)	1.17 (0.85–1.61)	1.44*** (1.32–1.57)	1.50*** (1.36–1.65)	0.93 (0.79–1.08)	1.06 (0.89–1.26)
**Type of earning from work**								
Not working	1.14** (1.06–1.24)	1.23*** (1.13–1.33)	0.92 (0.78–1.08)	0.86 (0.74–1.02)	0.98 (0.91–1.05)	1.06 (0.98–1.14)	1.11* (1.01–1.22)	1.13* (1.02–1.24)
Not paid	1.00 (0.88–1.14)	1.02 (0.9–1.16)	0.99 (0.76–1.3)	1.00 (0.76–1.32)	1.04 (0.95–1.15)	1.08 (0.98–1.19)	0.94 (0.84–1.05)	0.93 (0.83–1.04)
Paid^1^	***1*.*00 (Ref*.*)***	***1*.*00 (Ref*.*)***	***1*.*00 (Ref*.*)***	***1*.*00 (Ref*.*)***	***1*.*00 (Ref*.*)***	***1*.*00 (Ref*.*)***	***1*.*00 (Ref*.*)***	***1*.*00 (Ref*.*)***
**Exposure to media**								
No	1.12*** (1.07–1.18)	1.12*** (1.06–1.18)	0.98 (0.85–1.14)	0.99 (0.85–1.15)	1.02 (0.94–1.1)	1.05 (0.97–1.14)	1.10* (1.01–1.20)	1.11* (1.01–1.21)
Yes	***1*.*00 (Ref*.*)***	***1*.*00 (Ref*.*)***	***1*.*00 (Ref*.*)***	***1*.*00 (Ref*.*)***	***1*.*00 (Ref*.*)***	***1*.*00 (Ref*.*)***	***1*.*00 (Ref*.*)***	***1*.*00 (Ref*.*)***
**Household decision-making autonomy**								
Low	***1*.*00 (Ref*.*)***	***1*.*00 (Ref*.*)***	***1*.*00 (Ref*.*)***	***1*.*00 (Ref*.*)***	***1*.*00 (Ref*.*)***	***1*.*00 (Ref*.*)***	***1*.*00 (Ref*.*)***	***1*.*00 (Ref*.*)***
Medium	0.94* (0.89–0.99)	0.97 (0.92–1.02)	0.81* (0.69–0.95)	0.79** (0.67–0.94)	1.10* (1.01–1.20)	1.08 (0.99–1.17)	0.86** (0.79–0.94)	0.86*** (0.79–0.93)
High	1.21** (1.07–1.36)	1.30*** (1.15–1.46)	1.17 (0.86–1.60)	1.16 (0.84–1.58)	1.40** (1.14–1.72)	1.35** (1.09–1.66)	1.97*** (1.59–2.44)	1.94*** (1.56–2.4)
**Household wealth index**								
Poorest	1.18*** (1.08–1.28)	1.15** (1.05–1.26)	0.63*** (0.50–0.81)	0.62** (0.48–0.82)	0.87* (0.77–0.98)	0.96 (0.83–1.11)	1.43*** (1.23–1.67)	1.24* (1.04–1.48)
Poorer	1.15*** (1.07–1.24)	1.13** (1.04–1.22)	0.63*** (0.50–0.80)	0.63*** (0.48–0.81)	0.87* (0.77–0.98)	0.96 (0.84–1.11)	1.22** (1.05–1.42)	1.06 (0.89–1.25)
Middle	1.12** (1.04–1.21)	1.09* (1.01–1.18)	0.70** (0.55–0.88)	0.70** (0.55–0.91)	0.94 (0.84–1.06)	1.02 (0.90–1.17)	1.21* (1.04–1.41)	1.06 (0.90–1.26)
Richer	1.11** (1.03–1.2)	1.09* (1.01–1.17)	0.72** (0.57–0.91)	0.73* (0.58–0.93)	1.02 (0.91–1.15)	1.08 (0.96–1.22)	1.18* (1.01–1.36)	1.08 (0.92–1.26)
Richest	***1*.*00 (Ref*.*)***	***1*.*00 (Ref*.*)***	***1*.*00 (Ref*.*)***	***1*.*00 (Ref*.*)***	***1*.*00 (Ref*.*)***	***1*.*00 (Ref*.*)***	***1*.*00 (Ref*.*)***	***1*.*00 (Ref*.*)***
**Random effects parameter**	**Variance (SE)**							
Community level	0.18 (0.12)	0.16 (0.11)	0.09 (0.07)	0.07 (0.05)	0.11 (0.04)	0.09 (0.04)	0.14 (0.07)	0.14 (0.07)
Country level	0.47 (0.03)	0.49 (0.03)	0.23 (0.09)	0.22 (0.09)	0.26 (0.03)	0.26 (0.03)	0.26 (0.05)	0.25 (0.04)
**Intraclass correlation**	**ICC (SE)**							
Community level	0.17 (0.03)	0.17 (0.02)	0.09 (0.03)	0.08 (0.03)	0.1 (0.01)	0.1 (0.01)	0.11 (0.02)	0.11 (0.02)
Country level	0.05 (0.03)	0.04 (0.03)	0.03 (0.02)	0.02 (0.01)	0.03 (0.01)	0.03 (0.01)	0.04 (0.02)	0.04 (0.02)
**Likelihood-ratio test**								
Chi-square value	885.2	811.2	48.1	34.3	566.5	480.6	255.8	235.8
P value	<0.001	<0.001	<0.001	<0.001	<0.001	<0.001	<0.001	<0.001

N, number of total observations; CI, confidence interval; Ref., reference category; SE, standard error

^1^either cash, or in-kind, or both.

Model I included exposure variables only (educational level, type of earning, exposure to media, decision making autonomy, and household wealth index).

Model II was additionally adjusted with spousal age difference, partner more educated than wife, number of living children, married before 18 years old, and place of residence.

Significance level:

*p<0.05;

**p<0.01;

***p<0.001.

However, from the pooled data of 32 low- and lower-middle-income countries of Asia and SSA, the adjusted association revealed that primary education level, secondary and higher education level, not working status, no media exposure, high decision-making autonomy, poorest wealth index had positive association, whereas women’s medium decision-making autonomy showed negative but significant association with UNMC (S4 Table).

## 4. Discussion

To the best of our knowledge, this is the first comprehensive study that explored and compared the associated socioeconomic factors of younger married women with their UNMC, across the 32 LMICs of Asia and African region. Younger married women from SA region were ahead of their counterparts of SSA regions in terms of modern contraceptive usage. But UNMC was highly reported among the SA young married women followed by WCA, SEA, and ESA regions. Different socioeconomic factors of the study population like- higher educational level (in SA and WCA), not working status (in SA and ESA), no exposure to media (in SA and ESA), and higher decision-making autonomy (in SA, WCA, and ESA) showed positive and significant association with UNMC. Poorest households were positively associated with UNMC among the women of SA and ESA, whereas it showed negative association with UNMC in SEA and WCA regions.

Similar to our study, a recent investigation of World Family Planning (2017) indicated that SA had a higher rate of modern contraceptive use than Africa and SEA [22]. Again, UNMC was found to be higher where modern contraceptive prevalence was low [22], i.e., in WCA, SEA, and ESA regions; which is also similar to our study findings, except for SA. Younger married women hold new norms about family planning and family size due to the development of their empowerment status [19], which can outpace the availability and use of contraceptives [22]. This might be one of the plausible reasons behind the stable or increasing prevalence of UNMC among the younger married women of SA. On the contrary, WCA reported higher UNMC in our study which might be explained due to the high-level usage of traditional contraceptive methods [23], and unaware, unavailability, and cost of modern contraception [24]. Regionally, the women of Africa (Benin 36%, Burkina Faso 27%, Burundi 33%, Cameroon 33.2%, DR Congo 40%, Ghana 34%, Liberia 32%, and Uganda 33%) and Southern parts of Asia (Afghanistan 28%, Nepal 26%, Pakistan 30%, and Sri Lanka 22%) experienced more UNMC than other regions (Southeast Asia and East Asia < 20%) [6]; which was nearly consistent for younger married women of this study.

While exploring the associated socioeconomic factors, the UNMC of younger married women of SA, WCA, and ESA showed positive association with both higher educational level, and high decision-making autonomy. A comparative study of Kerry MacQuarrie conducted in 41 developing countries also reported similar positive relationship between higher educational attainment and UNMC [2], but this outcome is contradicted with the some studies from Pakistan [8], Ethiopia [14], and some African countries [9]. The possible reason behind such contradiction might be the age of study population. The study population of these aforementioned studies [8,9,14] included reproductive aged women (15–49 years), whereas our study as well as the study of Kerry MacQuarrie [2] considered only the younger married women. Even though young women can be educated and aware of contraceptive usage, factors like- pregnancy expectations early in marriage, male child preferences, limited access to modern spacing contraceptives (such as- oral contraceptive pills, intrauterine devices, condoms, and sterilizations, etc.), family resistance to adopt contraceptives, and husband’s reluctance on family planning issues etc. can increase their UNMC [25]. NGO conducted yard-meeting, counseling services from family planning workers, and teaching basic family planning education at schools etc. might be effective to eradicate the existing reluctance, resistance, and primitive misconceptions of using modern contraception among the spouses, and other family members.

Similar to our study, one of the studies from Southern Asia [19] showed that reproductive aged women (15–49 years) with higher decision-making autonomy used modern contraceptives frequently and experienced less UNMC. Women’s decision-making autonomy greatly depends on their age at marriage. Women marrying at a premature age usually possess lower social standing in the household, whereas later marriage provides a woman proper authority inside the home, ability to negotiate with household members, and strong involvement in decision-making after marriage [26]. So, in many LMICs, when younger women try to raise their voices, especially for their reproductive rights, and try to make a decision regarding her choice of using family planning methods, they experience different types of spousal violence [20]. On the other hand, couples possessing an equalitarian power structure at household and women holding medium level of authority within the home appeared to be more effective in satisfying their unmet contraception demand [27]. That is why, high decision-making authority showed positive association with UNMC among the married young women of SA, WCA, and ESA region in this study. By promoting community-based outreach campaigns and multisectoral programs for family planning focusing on couple’s egalitarian decision-making power structure in the household might reduce the level of UNMC [27].

In both SA and ESA, unemployment, and poorest wealth-index were positively associated with the experience of UNMC among the study population. These findings were accordant with some empirical study results [2,8,9,15]. Employed women, as well as, women from the economically advantaged household are usually able to increase their opportunity cost of bearing and rearing a child, compared to the unemployed and poor women [8]. But the scenario is different in most of the low-income countries. Because rearing babies by baby sitters are too much costly and they are not always available in LMICs [28]. In such cases, the mother’s sole responsibility of bearing and rearing a child reduces their time devoted to paid work and consequently, they may have to forego their source of income. Thus, unemployed and poor mothers try to avoid the extension of their family size and focus on the cost management of the household. Moreover, compared to the poor families, solvent households possess better access to modern contraceptives and most of the family planning services [8]. Therefore, similar to the studies of Nigeria, Pakistan, and Zambia [9], our study observed an increased likelihood of UNMC among the unemployed women and poor households, compared to the employed and rich ones. Introducing home-craft markets and promoting different micro-finance programs will create more employment opportunities for younger women. Establishing healthcare complex in remote and rural areas will provide better access to family planning services among the underprivileged population. Additionally, Non-Government organizations (NGOs) and local governing bodies should supply modern contraceptives at a low cost to the economically disadvantaged regions.

Consistent with previous studies [8,29], lack of exposure to family planning messages via media was found to be one of the major socio-economic determinants of UNMC in this study. The plausible reasons might be the lack of knowledge about the advantages of contraceptive usages, negative perceptions, and the excessive fear of side-effects of contraception [8,29,30]. A qualitative study from the rural areas of India [25] revealed that young married couples without proper media access have the misperception about the usage of oral contraceptive pills and intrauterine devices. For the last two decades, Governments of LMICs have been implementing a lot of actions to convince people concerning to the efficacy of birth control programs via extensive media campaign, where the messages of celebrities and influential personalities of the society are communicated to people to persuade them about the benefits of family planning programs. But, due to the lack of access to media among rural women, the effort of the government does not seem to be fruitful to change the perception of rural and superstitious people about the side effects of using contraceptives [8,30,31]. However, the access to media has to be increased through intervening programs, and family planning messages, advertisements, and campaigns via mass media should be accelerated. Such campaigns and messages may be helpful to remove the superstitions and fear of side-effects of using contraceptives among rural and lowly educated people. Additionally, this will eventually increase the awareness about their sexual and reproductive health, the acceptability of using the modern contraceptive, and the autonomy in fertility decision making.

The prime strength of this study comes from using the large and nationally representative surveys from 32 LMICs of Asia and Sub-Saharan Africa. So far, this is the first study that has estimated the country-wise UNMC among younger women as well as regionally examined and compared its association with women’s socio-economic status. Most importantly, this study was limited to those younger women who were married. Because, by including unmarried women, estimates in some regions may be underrepresented as they (some African and South Asian countries) have limited data on reproductive health for unmarried women, and many unmarried women with sexual experience may feel uncomfortable to report, which could potentially bias the measurements. On the other hand, the study sample was limited to only younger married women aged 15–24, while the data was mainly dependent on the verbal report provided by them. Again, women’s perception of wanting the next pregnancy or spacing it may change during the pregnancy, or depend on different circumstances of life. Additionally, the possibility of social desirability bias retains due to the self-reporting nature of the data collection with unknown validity and reproducibility. Finally, as this was a cross-sectional study, it was not possible to make any causal inference but rather only associations.

## 5. Conclusion

The highest coverage of modern contraceptive usage among the younger married women was reported in SA and the lowest was in WCA. But women from SA and ESA experienced the highest and lowest proportion of UNMC, respectively. In SA, women’s socioeconomic factors like- higher education, unemployment, lack of media accessibility, high decision-making autonomy, and poor wealth-index etc. showed positive association, whereas medium decision-making autonomy and poor wealth-index showed negative association with UNMC in SEA. High decision-making autonomy increased women’s UNMC in both WCA and ESA. Additionally, higher education in WCA, and unemployment, no media exposure, as well as poor wealth-index in ESA were positively associate with women’s experience of UNMC; which is a noteworthy contribution to the field of UNMC. However, to achieve Sustainable Development Goals (SDGs) target 3.7, i.e., ensuring universal access to sexual and reproductive health-care services by 2030, the international community must continue the existing campaigns of increasing modern contraceptive usages worldwide. And policy makers of respective LIMCs can implement versatile intervening program to reduce UNMC among the younger married women based on the findings and suggestions elicited in light of this comparative study.

## Supporting information

S1 Table Coverage of modern contraceptive usage and proportion of unmet need with modern contraceptives in 32 low- and middle-income countries. (DOCX)

S2 Table Socioeconomic status of study population by region. (DOCX)

S3 Table Bivariate association (percentage) of the socio-economic factors with unmet need for modern contraceptives in South Asia, Southeast Asia, West and Central Africa, and East and Southern Africa. (DOCX)

S4 Table Association of socio-economic factors with unmet need for modern contraceptives in 30 low- and middle-income countries (Pooled data). (DOCX)

## Abbreviations

AOR: Adjusted Odds Ratio
CI: Confidence Interval
DHS: Demographic and Health Survey
ESA: Eastern and Southern Africa
FP2020: Family Planning 2020
IRB: Institutional Review Board
LMICs: Low and Lower-Middle-Income Countries
SA: South Asia
SDGs: Sustainable Development Goals
SEA: Southeast Asia
SSA: Sub-Saharan Africa
UNMC: Unmet Need for Modern Contraception
WCA: West and Central Africa
