# Supplementary material for: Association between socioeconomic factors and unmet need for modern contraception among the young married women: A comparative study across the low- and lower-middle-income countries of Asia and Sub-Saharan Africa
Source: PLOS Glob Public Health. 2022 Jul 27;2(7):e0000731. doi: 10.1371/journal.pgph.0000731 (PMC10021951; doi:10.1371/journal.pgph.0000731)
Supplement: S1 Table — (DOCX) [file pgph.0000731.s001.docx]

**S1 Table.** Coverage of modern contraceptive usage and proportion of unmet need with modern contraceptives in 32 low- and middle-income countries (N=1,00,666)

| Country | Year | Survey | Percentage (95% confidence intervals) | |
| --- | --- | --- | --- | --- |
|  |  |  | Modern contraceptive | Unmet need of modern contraceptives |
| **South Asia (n = 55,953)** | |  | **44.7 (43.9-45.6)** | **24.6 (24.0-25.1)** |
| Afghanistan | 2015 | DHS VII | 13.2 (11.2-15.4) | 27.8 (26.0-29.7) |
| Bangladesh | 2017-18 | DHS VII | 48.2 (46.5-50.0) | 20.5 (19.2-21.9) |
| India | 2015-16 | DHS VII | 54.4 (53.4-55.3) | 23.5 (22.8-24.2) |
| Nepal | 2016 | DHS VII | 21.2 (19.0-23.5) | 41.5 (39.0-43.9) |
| Pakistan | 2017-18 | DHS VII | 11.6 (9.7-13.8) | 22.5 (20.2-25.1) |
| **Southeast Asia (n = 4,937)** | |  | **36.5 (34.8-38.3)** | **24.0 (22.6-25.5)** |
| Colombia | 2014 | DHS VII | 31.9 (29.4-34.6) | 26.0 (23.8-28.4) |
| Myanmar | 2015-16 | DHS VII | 58.1 (54.5-61.6) | 14.8 (12.2-17.7) |
| Philippines | 2017 | DHS VII | 43.5 (38.9-48.3) | 25.7 (21.8-29.9) |
| Timor-Leste | 2016 | DHS VII | 17.9 (15.2-21.0) | 28.5 (25.4-31.8) |
| **West and Central Africa (n = 23,491)** | | | **8.7 (8.2-9.2)** | **24.2 (23.5-25.0)** |
| Angola | 2015-16 | DHS VII | 9.0 (5.7-13.9) | 34.9 (28.3-42.2) |
| Benin | 2017-18 | DHS VII | 7.5 (6.3-9.0) | 37.3 (34.8-39.8) |
| Cameroon | 2018 | DHS VII | 12.2 (9.8-15.0) | 23.2 (20.2-26.4) |
| Chad | 2014-15 | DHS VII | 2.3 (1.8-3.1) | 24.6 (22.8-26.5) |
| Congo DR | 2013-14 | DHS VI | 5.3 (6.8-4.2) | 35.1 (32.0-38.2) |
| Ghana | 2014 | DHS VII | 19.7 (15.2-25.1) | 33.6 (28.3-39.4) |
| Guinea | 2018 | DHS VII | 8.6 (6.7-10.9) | 19.9 (17.9-22.2) |
| Liberia | 2019-20 | DHS VII | 15.2 (10.8-20.1) | 35.0 (26.2-44.5) |
| Mali | 2018 | DHS VII | 13.6 (11.8-15.6) | 21.3 (19.2-23.4) |
| Nigeria | 2018 | DHS VII | 6.1 (5.3-6.9) | 15.6 (14.5-16.8) |
| Senegal | 2019 | DHS VIII | 16.6 (14.2-19.3) | 20.5 (17.9-23.4) |
| Sierra Leon | 2019 | DHS VII | 16.1 (14.1-18.2) | 26.5 (23.9-29.2) |
| Togo | 2013-14 | DHS VI | 13.1 (10.6-16.2) | 39.5 (43.1-36.0) |
| **Eastern and Southern Africa (n = 16,285)** | | | **42.7 (41.6-43.8)** | **21.5 (20.7-22.4)** |
| Burundi | 2016-17 | DHS VII | 23.5 (20.4-26.8) | 28.8 (25.6-32.2) |
| Ethiopia | 2016 | DHS VII | 36.5 (32.6-40.5) | 18.7 (16.0-21.8) |
| Kenya | 2014 | DHS VII | 46.7 (43.4-49.9) | 22.8 (20.2-25.6) |
| Lesotho | 2014 | DHS VII | 51.9 (48.0-55.7) | 23.6 (20.6-26.9) |
| Malawi | 2015-16 | DHS VII | 50.6 (48.6-52.7) | 19.8 (18.3-21.5) |
| Rwanda | 2014-15 | DHS VII | 43.1 (37.1-49.3) | 19.9 (15.3-25.5) |
| Tanzania | 2015-16 | DHS VII | 24.1 (21.2-27.2) | 25.7 (23.1-28.4) |
| Uganda | 2016 | DHS VII | 28.1 (25.2-31.3) | 32.6 (29.8-35.5) |
| Zambia | 2018 | DHS VII | 45.3 (42.3-48.4) | 20.6 (18.2-23.3) |
| Zimbabwe | 2015 | DHS VII | 58.5 (54.9-61.9) | 11.0 (9.2-13.1) |
| **Pooled total** |  |  | **35.6 (35.1-36.1)** | **24.0 (23.6-24.4)** |

N, number of observations;
