# Supplementary material for: Association between socioeconomic factors and unmet need for modern contraception among the young married women: A comparative study across the low- and lower-middle-income countries of Asia and Sub-Saharan Africa
Source: PLOS Glob Public Health. 2022 Jul 27;2(7):e0000731. doi: 10.1371/journal.pgph.0000731 (PMC10021951; doi:10.1371/journal.pgph.0000731)
Supplement: S2 Table — (DOCX) [file pgph.0000731.s002.docx]

**S2 Table.** Percentage distribution of the socio-economic status of study population by region

| **Characteristics** | **Percentage (95% Confidence Interval)** | | | | |
| --- | --- | --- | --- | --- | --- |
|  | **Pooled** | **South Asia** | **Southeast Asia** | **West and Central Africa** | **East and southern Africa** |
| **Educational level** |  |  |  |  |  |
| No education | 26.8 (26.3-27.4) | 23.0 (22.3-23.8) | 8.6 (7.5-9.9) | 50.3 (49.2-51.4) | 11.5 (10.5-12.5) |
| Primary | 24.2 (23.8-24.6) | 14.8 (14.2-15.3) | 34.1 (32.2-36.1) | 21.6 (20.9-22.4) | 57.5 (56.3-58.7) |
| Secondary and higher | 48.9 (48.4-49.5) | 62.2 (61.4-63.0) | 57.2 (55.1-59.3) | 28.1 (27.2-29.0) | 31.1 (29.8-32.3) |
| **Type of earning from work** |  |  |  |  |  |
| Not working | 66.8 (66.4-67.3) | 86.8 (86.3-87.4) | 42.6 (40.4-44.8) | 42.2 (41.1-43.2) | 41.0 (39.8-42.3) |
| Not paid | 10.6 (10.2-10.9) | 4.25 (3.9-4.7) | 7.0 (6.1-8.0) | 16.3 (15.5-17.1) | 25.2 (23.9-26.3) |
| Paid^1^ | 22.6 (22.2-23.0) | 8.9 (8.5-9.3) | 50.5 (48.2-52.7) | 41.5 (40.5-42.5) | 33.9 (32.8-35.1) |
| **Exposure to media** |  |  |  |  |  |
| No | 54.8 (54.2-55.3) | 45.8 (45.0-46.6) | 55.2 (53.1-57.4) | 73.9 (73.1-74.8) | 57.8 (56.5-59.0) |
| Yes | 45.2 (44.7-45.8) | 54.2 (53.4-55.0) | 44.8 (42.7-46.9) | 26.1 (25.2-26.9) | 42.3 (41.0-43.5) |
| **Household decision making autonomy** | |  |  |  |  |
| Low | 50.8 (50.3-51.3) | 40.3 (39.5-41.1) | 26.2 (24.5-27.9) | 80.4 (79.6-81.2) | 51.6 (50.5-52.7) |
| Medium | 46.2 (45.7-46.7) | 56.2 (55.4-57.0) | 68.5 (66.7-70.2) | 17.8 (17.0-18.5) | 46.0 (44.8-47.1) |
| High | 3.0 (2.9-3.2) | 3.5 (3.2-3.7) | 5.4 (4.6-6.2) | 1.8 (1.6-2.1) | 2.4 (2.1-2.8) |
| **Household wealth index** |  |  |  |  |  |
| Poorest | 20.3 (19.9-20.8) | 17.8 (17.1-18.4) | 23.6 (21.7-25.6) | 23.1 (22.1-24.2) | 24.0 (22.9-25.2) |
| Poorer | 23.2 (22.8-23.7) | 22.7 (22.1-23.3) | 20.9 (19.4-22.5) | 24.5 (23.6-25.4) | 23.7 (22.7-24.8) |
| Middle | 21.6 (21.2-22.0) | 22.8 (22.2-23.5) | 20.6 (19.0-22.3) | 20.6 (19.8-21.5) | 19.1 (18.2-20.0) |
| Richer | 20.0 (19.6-20.5) | 21.2 (20.6-21.9) | 19.5 (17.8-21.4) | 18.1 (17.3-18.9) | 18.8 (17.8-19.9) |
| Richest | 14.9 (14.5-15.3) | 15.5 (14.9-16.1) | 16.4 (13.8-17.1) | 13.7 (13.0-14.5) | 14.4 (13.4-15.5) |

^1^either cash, or in-kind, or both;
