# Supplementary material for: Association between socioeconomic factors and unmet need for modern contraception among the young married women: A comparative study across the low- and lower-middle-income countries of Asia and Sub-Saharan Africa
Source: PLOS Glob Public Health. 2022 Jul 27;2(7):e0000731. doi: 10.1371/journal.pgph.0000731 (PMC10021951; doi:10.1371/journal.pgph.0000731)
Supplement: S3 Table — (DOCX) [file pgph.0000731.s003.docx]

**S3 Table.** Bivariate association (percentage) of the socio-economic factors with unmet need for modern contraceptives in South Asia, Southeast Asia, West and Central Africa, and East and Southern Africa

| **Socio-economic factors** | **Unmet need for modern contraceptives,**  **% (95% CI)** | | | | | | | | | |
| --- | --- | --- | --- | --- | --- | --- | --- | --- | --- | --- |
|  | **Pooled**  (N = 1,00,666) | ***P-values* (ꭓ2)** | **South Asia**  (N = 55,953) | ***P-values* (ꭓ2)** | **Southeast Asia**  (N = 4,937) | ***P-values* (ꭓ2)** | **West and Central Africa** (N = 23,491) | ***P-values* (ꭓ2)** | **East and Southern Africa** (N = 16,285) | ***P-values* (ꭓ2)** |
| **Educational level** | | *P = 0.001* |  | *P = 0.149* |  | *P = 0.421* |  | *P < 0.001* |  | *P < 0.001* |
| No education | 22.8 (22.1-23.5) |  | 25.1 (24.0-26.2) |  | 21.4 (16.9-26.6) |  | 20.0 (19.1-20.9) |  | 24.6 (22.1-27.3) |  |
| Primary | 24.3 (23.5-25.0) |  | 23.4 (22.1-24.7) |  | 23.5 (21.0-26.2) |  | 28.0 (26.5-29.6) |  | 23.1 (22.0-24.3) |  |
| Secondary and higher | 24.5 (23.9-25.1) |  | 24.6 (23.9-25.4) |  | 24.7 (22.9-26.7) |  | 28.9 (27.6-30.3) |  | 17.5 (16.2-18.9) |  |
| **Type of earning from work** | | *P = 0.214* |  | *P < 0.001* |  | *P = 0.489* |  | *P < 0.001* |  | *P = 0.270* |
| Not working | 23.8 (23.3-24.3) |  | 24.6 (23.9-25.2) |  | 23.7 (21.5-26.1) |  | 22.0 (21.0-23.0) |  | 20.9 (19.6-22.1) |  |
| Not paid | 24.8 (23.8-25.8) |  | 29.1 (26.9-31.5) |  | 27.2 (22.3-32.8) |  | 25.3 (23.7-27.0) |  | 21.5 (19.9-23.2) |  |
| Paid^1^ | 24.1 (23.4-24.9) |  | 22.4 (20.8-24.1) |  | 23.9 (21.8-26.1) |  | 26.1 (25.0-27.3) |  | 22.4 (21.1-23.8)) |  |
| **Exposure to media** | | *P = 0.278* |  | *P = 0.007* |  | *P = 0.121* |  | *P = 0.026* |  | *P = 0.276* |
| No | 24.2 (23.7-24.7) |  | 25.4 (24.6-26.2) |  | 22.9 (21.1-25.0) |  | 23.8 (23.0-24.6) |  | 21.9 (20.9-23.1) |  |
| Yes | 23.7 (23.1-24.3) |  | 23.9 (23.1-24.7) |  | 25.3 (23.1-27.6) |  | 25.6 (24.2-27.0) |  | 21.0 (19.8-22.3) |  |
| **Household decision-making**  **autonomy** | | *P < 0.001* |  | *P < 0.001* |  | P = 0.349 |  | *P < 0.001* |  | *P < 0.001* |
| Low | 24.7 (24.1-25.2) |  | 26.4 (25.6-27.3) |  | 24.8 (22.0-27.8) |  | 23.1 (22.4-23.9) |  | 23.2 (22.1-24.4) |  |
| Medium | 22.8 (22.2-23.4) |  | 23.0 (22.3-23.8) |  | 23.4 (21.7-25.3) |  | 28.1 (26.4-29.9) |  | 18.7 (17.6-19.8) |  |
| High | 30.3 (28.0-32.6) |  | 27.5 (24.8-30.5) |  | 27.9 (22.1-34.4) |  | 35.2 (30.0-40.8) |  | 39.7 (22.1-46.8) |  |
| **Household wealth-index** | | *P = 0.997* |  | *P = 0.206* |  | *P = 0.036* |  | *P < 0.001* |  | *P < 0.001* |
| Poorest | 24.0 (23.2-24.8) |  | 25.4 (24.2-26.6) |  | 22.6 (19.9-25.5) |  | 21.3 (20.0-22.7) |  | 24.6 (23.0-26.3) |  |
| Poorer | 23.9 (23.2-24.7) |  | 25.4 (24.3-26.5) |  | 22.8 (19.9-25.9) |  | 21.9 (20.6-23.2) |  | 22.7 (20.9-24.6) |  |
| Middle | 23.9 (23.1-24.7) |  | 24.2 (23.1-25.3) |  | 24.3 (20.8-28.2) |  | 24.8 (23.4-26.4) |  | 21.2 (19.5-22.9) |  |
| Richer | 24.1 (23.2-25.1) |  | 23.9 (22.6-25.3) |  | 22.5 (19.3-26.0) |  | 28.1 (26.4-29.9) |  | 19.9 (18.0-21.9) |  |
| Richest | 23.9 (22.9-25.0) |  | 23.9 (22.4-25.4) |  | 29.6 (25.8-33.7) |  | 27.5 (25.5-29.5) |  | 17.2 (15.3-19.4) |  |

N, number of total observations; CI, confidence interval; *P-values* (ꭓ2), P values obtained from chi-square estimation;

^1^either cash, or in-kind, or both;
