# Supplementary material for: Association between socioeconomic factors and unmet need for modern contraception among the young married women: A comparative study across the low- and lower-middle-income countries of Asia and Sub-Saharan Africa
Source: PLOS Glob Public Health. 2022 Jul 27;2(7):e0000731. doi: 10.1371/journal.pgph.0000731 (PMC10021951; doi:10.1371/journal.pgph.0000731)
Supplement: S4 Table — (DOCX) [file pgph.0000731.s004.docx]

**S4 Table.** Association of socio-economic factors with unmet need for modern contraceptives in 30 low- and middle-income countries (Pooled data)

| **Socio-economic factors** | **Odds ratio (95% CI)** | |
| --- | --- | --- |
|  | **Model I** | **Model II** |
| **Educational level** | |  |
| No education | ***1.00 (Ref.)*** | ***1.00 (Ref.)*** |
| Primary | 1.15*** (1.09-1.20) | 1.15*** (1.10-1.21) |
| Secondary and higher | 1.26*** (1.20-1.32) | 1.38*** (1.31-1.46) |
| **Type of earning from work** | |  |
| Not working | 1.05* (1.01-1.10) | 1.09*** (1.04-1.14) |
| Not paid | 0.98 (0.93-1.05) | 1.00 (0.94-1.06) |
| Paid^1^ | ***1.00 (Ref.)*** | ***1.00 (Ref.)*** |
| **Exposure to media** | |  |
| No | 1.10*** (1.06-1.14) | 1.10*** (1.06-1.14) |
| Yes | ***1.00 (Ref.)*** | ***1.00 (Ref.)*** |
| **Household decision making autonomy** | |  |
| Low | ***1.00 (Ref.)*** | ***1.00 (Ref.)*** |
| Medium | 0.95** (0.91-0.98) | 0.92*** (0.89-0.96) |
| High | 1.35*** (1.24-1.48) | 1.33*** (1.22-1.46) |
| **Household wealth index** | |  |
| Poorest | 1.11*** (1.05-1.18) | 1.10** (1.03-1.17) |
| Poorer | 1.07* (1.01-1.13) | 1.05 (0.98-1.11) |
| Middle | 1.07* (1.01-1.13) | 1.05 (0.99-1.12) |
| Richer | 1.08** (1.02-1.14) | 1.07* (1.01-1.13) |
| Richest | ***1.00 (Ref.)*** | ***1.00 (Ref.)*** |
| **Random effects parameter: variance (SE)** | |  |
| Community level | 0.16 (0.04) | 0.16 (0.04) |
| Country level | 0.39 (0.02) | 0.39 (0.02) |
| **ICC (SE)** |  |  |
| Community level | 0.14 (0.01) | 0.14 (0.01) |
| Country level | 0.04 (0.01) | 0.04 (0.01) |
| **LR test** |  |  |
| Chi-square value | 2087.6 | 1923.9 |
| P value | <0.001 | <0.001 |

N, number of total observations; CI, confidence interval; Ref., reference category; SE, standard error

^1^either cash, or in-kind, or both;

Model I included exposure variables only (educational level, type of earning, exposure to media, decision making autonomy, and household wealth index)

Model II was additionally adjusted with spousal age difference, partner more educated than wife, number of living children, married before 18 years old, and place of residence

Significance level: *p<0.05; **p<0.01; ***p<0.001;
